# Supplementary material for: Halichoeres sanchezi n. sp., a new wrasse from the Revillagigedo Archipelago of Mexico, tropical eastern Pacific Ocean (Teleostei: Labridae)
Source: PeerJ. 2024 Feb 28;12:e16828. doi: 10.7717/peerj.16828 (PMC10908266; doi:10.7717/peerj.16828)
Supplement: Supplemental Information 1 — FASTA format nucleotide sequences for Halichoeres sanchezi (accession number OQ922018) and Halichoeres burekae (accession numbers OR588066 & OR588068). [file peerj-12-16828-s001.docx]

FASTA sequences for three Halichoeres species cited in Victor et al. (in review)

>Halichoeres sanchezi|gnl|uoguelph|LIDMA3845-22.COI-5P
CCTCTATTTAGTATTCGGTGCCTGAGCTGGGATAGTAGGCACAGCTTTAAGCCTGCTCATTCGAGCTGAGCTGAGCCAGCCCGGCGCTCTTCTCGGAGACGACCAAATCTATAATGTAATCGTCACTGCCCACGCCTTTGTAATAATTTTCTTTATAGTAATACCAATTATGATTGGCGGGTTCGGGAACTGACTAATTCCCCTTATGATTGGCGCACCCGACATAGCCTTCCCTCGAATGAACAACATGAGTTTCTGACTACTCCCCCCTTCTTTCCTTCTTCTCCTAGCCTCTTCTGGCGTAGAAGCAGGTGCTGGAACTGGCTGAACAGTTTACCCCCCGCTAGCAGGAAATTTAGCTCACGCTGGTGCATCTGTAGATCTTACGATCTTCTCCCTCCACTTAGCTGGAATTTCTTCAATCCTAGGTGCAATTAACTTTATTACAACTATTATTAACATAAAACCCCCCGCCATCTCTCAGTACCAAACACCTTTATTTGTCTGAGCGGTGTTGATTACAGCTGTACTTCTTCTCCTGTCACTACCCGTCCTTGCTGCAGGCATTACAATGCTCCTGACAGACCGAAACCTGAATACTACCTTCTTCGACCCCGCTGGGGGAGGAGACCCTATCCTATACCAGCACCTA

>Halichoeres burekae|gnl|uoguelph|LIDMA3677-22.COI-5P
CCTTTATCTAGTATTCGGCGCCTGAGCTGGGATAGTAGGTACGGCCTTGAGCCTACTTATTCGGGCTGAATTAAGCCAACCCGGCGCTCTCCTTGGGGACGACCAGATCTATAACGTAATCGTTACAGCCCATGCGTTCGTAATAATTTTCTTTATAGTAATACCAATTATGATCGGCGGATTTGGAAATTGACTAATCCCCCTCATGGTTGGTGCCCCTGACATGGCCTTCCCTCGAATAAATAATATGAGCTTTTGACTTCTCCCGCCTTCCTTCCTACTCCTGCTTGCCTCTTCTGGGGTAGAGGCTGGAGCCGGTACCGGATGAACAGTTTACCCCCCTTTAGCAGGTAATCTTGCACACGCCGGTGCATCTGTAGACCTCACAATCTTTTCTCTCCACCTGGCCGGTATTTCGTCGATTCTTGGGGCTATCAACTTCATTACTACTATTGTTAATATGAAACCCCCTGCTATTTCCCAATATCAAACACCCCTCTTCGTCTGAGCCGTCCTAATTACGGCCGTACTTCTCCTCCTTTCCCTCCCAGTGCTTGCCGCTGGTATTACAATGCTTCTTACAGATCGAAATTTAAATACTACTTTCTTTGACCCAGCTGGAGGGGGCGATCCCATCCTATACCAGCACTTA

>Halichoeres burekae|gnl|uoguelph|LIDMA3725-22.COI-5P
CCTTTATCTAGTATTCGGCGCCTGAGCTGGGATAGTAGGTACGGCCCTGAGCCTACTTATTCGGGCTGAATTAAGCCAACCCGGCGCTCTCCTTGGGGACGACCAGATCTATAACGTAATCGTTACAGCCCATGCGTTCGTAATAATTTTCTTTATAGTAATACCAATTATGATCGGCGGATTTGGAAATTGACTAATCCCCCTCATGGTTGGTGCCCCTGACATGGCCTTCCCTCGAATAAATAATATGAGCTTTTGACTTCTCCCGCCTTCCTTCCTACTCCTGCTTGCCTCTTCTGGGGTAGAGGCTGGAGCCGGTACCGGATGAACAGTTTACCCCCCTTTAGCAGGTAATCTTGCGCACGCCGGTGCATCTGTAGACCTCACAATCTTTTCTCTCCACCTGGCCGGTATTTCGTCGATTCTTGGGGCTATCAACTTCATTACTACTATTGTTAATATGAAACCCCCTGCTATTTCCCAATATCAAACACCCCTCTTCGTCTGAGCCGTCCTAATTACGGCCGTACTTCTCCTCCTTTCCCTCCCAGTGCTTGCCGCTGGTATTACAATGCTTCTTACAGATCGAAATTTAAATACTACTTTCTTTGACCCAGCTGGAGGGGGCGATCCCATCCTATACCAGCACTTA
